# Improved selectivity from a wavelength addressable device for wireless stimulation of neural tissue

**DOI:** 10.3389/fneng.2014.00005

**Published:** 2014-02-18

**Authors:** Elif Ç. Seymour, David S. Freedman, Mutlu Gökkavas, Ekmel Özbay, Mesut Sahin, M. Selim Ünlü

**Affiliations:** ^1^Department of Biomedical Engineering, Boston University, BostonMA, USA; ^2^Department of Electrical and Computer Engineering, Boston University, BostonMA, USA; ^3^Nanotechnology Research Center, Bilkent UniversityAnkara, Turkey; ^4^Department of Biomedical Engineering, New Jersey Institute of Technology, NewarkNJ, USA

**Keywords:** optical neural stimulation, addressable stimulators, optically powered, wireless, floating micro electrodes, neural prostheses

## Abstract

Electrical neural stimulation with micro electrodes is a promising technique for restoring lost functions in the central nervous system as a result of injury or disease. One of the problems related to current neural stimulators is the tissue response due to the connecting wires and the presence of a rigid electrode inside soft neural tissue. We have developed a novel, optically activated, microscale photovoltaic neurostimulator based on a custom layered compound semiconductor heterostructure that is both wireless and has a comparatively small volume (<0.01 mm^3^). Optical activation provides a wireless means of energy transfer to the neurostimulator, eliminating wires and the associated complications. This neurostimulator was shown to evoke action potentials and a functional motor response in the rat spinal cord. In this work, we extend our design to include wavelength selectivity and thus allowing independent activation of devices. As a proof of concept, we fabricated two different microscale devices with different spectral responsivities in the near-infrared region. We assessed the improved addressability of individual devices via wavelength selectivity as compared to spatial selectivity alone through on-bench optical measurements of the devices in combination with an *in vivo* light intensity profile in the rat cortex obtained in a previous study. We show that wavelength selectivity improves the individual addressability of the floating stimulators, thus increasing the number of devices that can be implanted in close proximity to each other.

## INTRODUCTION

Intraspinal micro stimulation is a technique of electrically activating the neuronal networks in the spinal segments that have lost their innervation from the proximal cord as a result of injury (e.g., spinal cord injury) or disease, e.g., amyotrophic lateral sclerosis (ALS), and other forms of demyelination in the central nervous system (CNS). Examples of functions that may be restored by spinal cord micro stimulation include respiration, bowel/bladder activity, and some components of upper or lower limb functions (Hamid and Hayek, 2008). A neural prosthetic device activates the neurons extracellularly by injecting electrical charge into the neural tissue. Injection current depolarizes nearby cell membranes, thus triggering an action potential that in turn is synaptically transmitted to other neurons and finally to the muscles to restore the lost motor movements.

Traditional electrodes suffer from a number of complications. During insertion, needle-like electrodes induce mechanical trauma because the insertion path of the electrode passes through the capillaries, extracellular matrix, glial, and neuronal cell processes (Polikov et al., 2005). A micrometer-scale stimulator that is free of wires would decrease the amount of tissue displaced by the stimulator and hence decrease the mechanical trauma of insertion and the cellular response due to the shear forces imposed on the tissue surrounding the implant. Moreover, chronic tissue response is increased by wires and associated tethering forces (Biran et al., 2007). Mounting problems and wire breakage are also difficulties that lead to device failure (Hetke et al., 1994). Thus, there is increasing interest in using wireless stimulation in the neural prosthesis community as a method to eliminate problematic wires. For example, researchers have utilized pulsed ultrasound as a wireless energy transfer to stimulate brain circuits (Tufail et al., 2010). Additionally, radio-frequency radiation is seen as a potential wireless paradigm for transferring power and data, but it requires the use of a coil that is typically in the millimeter size range (Harrison et al., 2007). Current wireless stimulation paradigms are reviewed by Sahin and Pikov (2011).

In addition to being wireless, a practical neurostimulator should also be individually addressable. The complexity of the nervous system requires very local activation of neurons in the CNS. New approaches have been developed to address the spatial selectivity issue. Optogenetic stimulation is a method that involves genetic manipulation of nerve cells to express a light responsive ion channel (Aravanis et al., 2007). Although optogenetic stimulation provides addressability through spatial selectivity, the depth of light penetration is limited to a few hundred micrometers at the visible wavelengths that are commonly used. Additionally, the safety of long-term protein expression in the human brain needs further research before translation of this technology to the clinic. Our search for a neurostimulation microsystem that is small, selective, and wireless has led us to target semiconductor photovoltaic devices for harvesting the optical activation signal sent into the neural tissue and converting that signal into electric current. Previously, it has been shown that a microscale photovoltaic neurostimulator can activate peripheral nerves when illuminated with a laser source at 852 nm through an optical fiber (Song et al., 2007). However, this system does not offer a solution to the mechanical stress associated with tethered connections because it uses an optical fiber attached to the photovoltaic device as the means of energy transfer.

As a solution to the aforementioned problems, we have developed a novel wireless, floating, and individually addressable neurostimulation device that can be optically activated. The proposed floating light activated micro-electrical (FLAME) stimulators use wavelength-specific light to energize the device and therefore have the potential of activating neural cells with spatial selectivity based on the wavelength of light being used. Moreover, because the FLAME stimulator (FLAMES) does not contain active circuits that need to be powered continuously, chronic implants that do not require a battery are possible. The FLAME stimulators are designed to work in the near-infrared (NIR) region because of the high conversion efficiency of photovoltaic devices and low-absorption of light in the tissue at these wavelengths (Weissleder, 2001). It was demonstrated that FLAME stimulators are able to generate sufficient currents to evoke a functional response in the rat spinal cord (Abdo et al., 2011). However, spatial and wavelength selectivity of FLAME stimulators were not evaluated in that study.

A wavelength selective photodiode can be fabricated using different approaches: (1) a Fabry-Perot interferometer; (2) a diffraction grating; (3) resonant cavity enhanced (RCE) photodetectors; (4) filterless photodetector; (5) passive optical block. It is not feasible to use all these approaches in a practical neurostimulator.

A Fabry-Perot interferometer is an optical element, consisting of two partially reflecting parallel mirrors separated by a gap, which can be used as a wavelength selective filter (Jerman and Clift, 1991). Resonant transmission peaks occur when the gap equals integer multiples of half the wavelength of the incident light. A photodetector with an integrated resonant cavity was fabricated and characterized (Hunt et al., 1993). In addition, a deformable membrane interferometer device was developed with a coupled air/semiconductor optical cavity into which a photodiode could be incorporated (Larson et al., 1995). This method does not lend itself to our application since the transmission of a Fabry-Perot filter is a function of the angle of incidence of the beam. Wavelength selective characteristics of the filter would change as the incident light deviates from the normal, as is the case in tissue.

A diffraction grating, another method of implementing wavelength selectivity, is commonly used for dispersion of the spectrum of incident radiation into different wavelength components (Kong et al., 2001). However, in a grating, the diffraction pattern changes as a function of the angle of the incident light, which cannot be controlled in tissue (Kwa and Wolffenbuttel, 1992). Resonant cavity-enhanced photodetectors are another realization of wavelength selective photonic devices, where the device performance is enhanced by placing the active device structure inside a Fabry-Perot resonant microcavity (Unlu and Strite, 1995). The quantum efficiency of a resonant cavity photodetector also depends on the angle of incident light. When the incident angle is shifted from the normal, the peak value of quantum efficiency will decrease since the change in the optical path length causes a shift from the resonance wavelength (Kishino et al., 1991). Wavelength selective photodetectors mentioned so far are not suitable for use in an implantable neurostimulator because intense scattering takes place in tissue and the angle of light incident on the photodetector cannot be controlled.

There has also been effort to create a filterless photodetector that can select narrow bands in the optical spectrum (Khudaverdyan et al., 2007). A multi-band spectroscopic photodetector array structure was introduced where different photodetection elements use different thicknesses to govern the wavelength of the detection elements (Kalkhoran and Namavar, 1997). Although these photodetectors are independent of the angle of incidence, they require electronic readout circuitry to change the external bias voltage, making them impractical for use in a fully implantable micro stimulator. Therefore, we use the passive optical block method to achieve wavelength selectivity.

## MATERIALS AND METHODS

### WAVELENGTH SELECTIVITY

Our group has previously developed silicon semiconductor photodetector devices that can function as optically activated micro stimulators. Although it was demonstrated that silicon photodiodes are feasible for wireless neurostimulation (Abdo et al., 2009), we switched to III–V semiconductor (Al_x_Ga_(1-x)_As) photodiodes for our current study because of three key advantages. First, simulations of thin film filters deposited on the top surface of silicon showed that the resonant wavelength of the interference filter varies with the angle of illumination and therefore wavelength selectivity could not be integrated into silicon devices. Second, because GaAs semiconductors are direct bandgap devices, they have significantly larger absorption coefficients than silicon in NIR wavelengths; therefore, they have higher quantum efficiencies that lead to increased optical to electrical conversion efficiencies. The increased quantum efficiency, as compared to silicon devices, translates into lower optical power requirements for activation of the stimulators. Also, because the GaAs semiconductors have a larger bandgap voltage, the device size can further be reduced and a more local activation can be produced. Finally, an important benefit of using III–V semiconductor technology is that series photodiodes are easily made through vertically integrated heterojunctions, which contrasts with planar silicon semiconductor devices.

**FIGURE 1:**
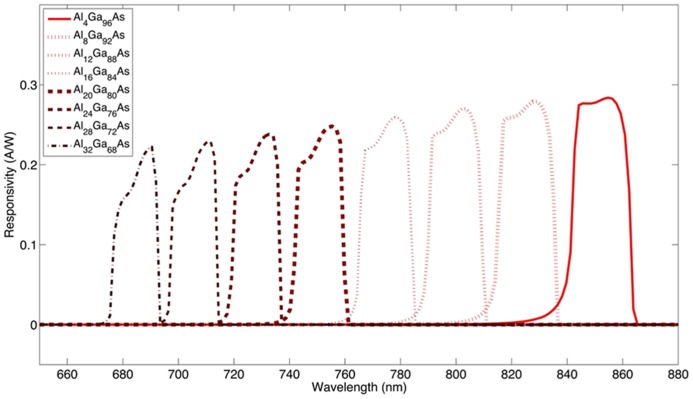
**Simulation of a class of wavelength selective devices.** The optical block aluminum concentration was increased in 4% increments relative to the p-i-n diode, which was at lower aluminum concentrations.

In our custom layered Al_x_Ga_(1-x)_As photodetector structures, wavelength selectivity is achieved by adjusting the proportional composition of aluminum and gallium in the GaAs/AlGaAs compound semiconductor system. The ability to modify the optical characteristics of compound semiconductor heterostructures by adjusting the compositional variation is known as bandgap engineering. A large bandgap layer is utilized as an optical block to filter shorter wavelengths and the active layer of the photodiode is engineered to limit responsivity to specific wavelengths. An example of using bandgap engineering to create a series of individually addressable devices is shown in **Figure 1**. As a proof of concept, we have designed two different custom layered GaAs/Al_x_Ga_(1-x)_As heterostructures providing two different stimulation channels with their corresponding wavelengths in the NIR region. In this work, we present simulations of expected spatial and wavelength selectivity of our devices when they are activated by wavelength specific light inside the rat brain cortex.

### STRUCTURE OF GaAs/Al_x_Ga(_1-x_)As WAFERS

**FIGURE 2:**
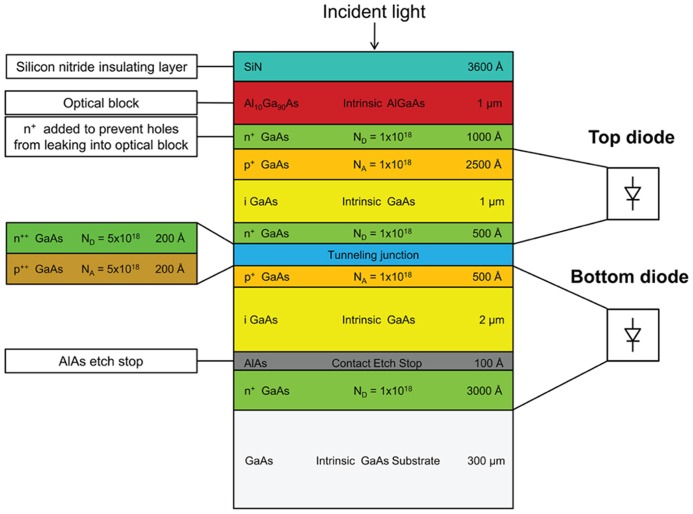
**Cross-sectional schematic of one of the two wafer structures for FLAME stimulators.** This particular structure consists of an optical block and two series photodiodes connected through a highly doped tunneling junction. This structure (FLAMES A) is designed to provide maximum responsivity around 860 nm.

Our photovoltaic structures are based on custom layered compound semiconductor heterostructures utilizing gallium arsenide/aluminum gallium arsenide (GaAs/Al_x_Ga_(1-x)_As) on a gallium arsenide substrate. The multilayered GaAs/Al_x_Ga_(1-x)_As wafers were grown by ITME (Institute of Electronic Materials Technology, Poland). The wafers have eleven epitaxial layers consisting of two p-i-n junctions that are vertically connected through a highly doped tunneling junction region. Two diodes are connected in series to increase the open-circuit voltage across the diode and reduce the requirements on the contact resistance and thus require less optical power to induce neurostimulation. The layer thicknesses are optimized for matching the current in the upper and lower cells and also to provide reasonable etch depths. The top layer serves as an optical block to filter shorter wavelengths. Two custom wafer structures are fabricated, each having a different Al_x_Ga_(1-x)_As composition in the optical block and photodiode layers: one with a composition of Al_0.1_Ga_0.9_As as the optical block and GaAs as the photodiode layer, the other with a composition of Al_0.2_Ga_0.8_As as the optical block and Al_0.1_Ga_0.9_As as the photodiode layer. The former structure, shown in **Figure 2**, is designed to have a peak responsivity around 860 nm and the latter was designed to have a peak responsivity around 780 nm. Two different spectral responsivities are used to demonstrate the wavelength addressability. Throughout the paper, these devices will be referred to as FLAME A, and FLAME B stimulators, respectively. Aluminum concentration values were specified when requesting the wafers, however, as it will be explained in the results sections, actual aluminum concentrations were found to differ from these specified values. The designed aluminum concentration of the layers was optimized to provide a good compromise between responsivity and wavelength selectivity. We performed quantum efficiency simulations of the wafer structures with a custom MATLAB program that calculates the reflection and quantum efficiency from a layered structure using film scattering matrix calculations. Real and imaginary values of the refractive index for intermediate aluminum concentrations were calculated based on model functions by Adachi (1985).

### MICRODEVICE FABRICATION

**FIGURE 3:**
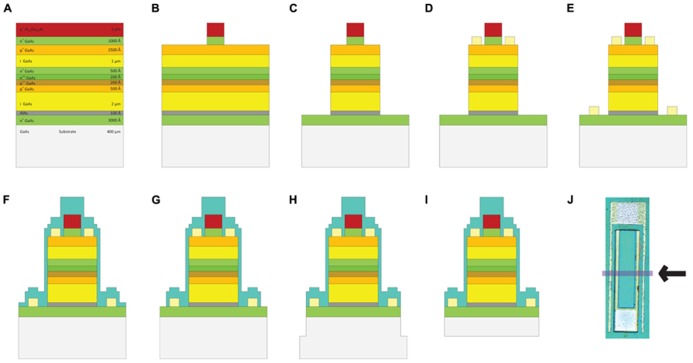
**Fabrication steps of GaAs FLAME stimulators (A) GaAs/AlGaAs wafer structure, (B) etching down to the p-layer, (C) etching down to n-layer, (D) p metal deposition: Ti:Au, (E) n-metal deposition: Ge:Au:Ge:Au:Ni:Au, (F) silicon nitride (SiN) deposition, (G) etching SiN from the contacts, (H) top release: chips are diced 150 μm-deep around the devices, (I) backside etching to complete the release of devices, (J) the micrograph of a completed FLAME device with the arrow indicating the cross section shown in (A–I)**.

**FIGURE 4:**
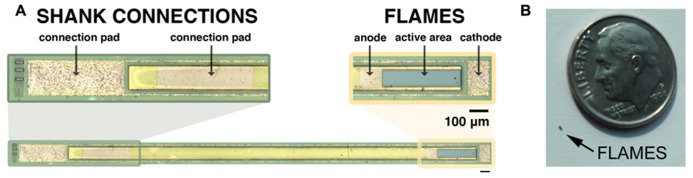
**(A)** Micrograph of a completed shank device. Shanks are longitudinal structures with the device on one end and connection pads on the other end for acute *in vivo* testing. Image was zoomed into two ends: left image shows connection pads at the end of the shank. Right image shows device end of the shank with its anode, cathode and active area (scale bar is 100 μm). **(B)** Photograph of FLAME device next to a USA dime coin.

Two-inch diameter wafers were diced into 9 mm × 10 mm pieces with the DISCO automatic dicer and the layer structures were verified with scanning electron microscopy (Zeiss). Fabrication consisted of standard positive photolithography, wet and dry etching, metallization and passivation steps, which are shown in **Figure 3**. Mask layouts were drawn using the Virtuoso tool (Cadence). Mask designs were transferred to iron oxide masks with the DWL 66 mask writer (Heidelberg Instruments). Series photodiodes were implemented by making the p-contact on the p-type layer of the first (top) photodiode and the n-contact on the n-type layer of the second (bottom) photodiode. Two series photodiodes were connected to each other through a highly doped tunneling junction. The solution for etching to the p-type layer was prepared by mixing 25:5:1 (volume):deionized (DI) H_2_O:phosphoric acid:H_2_O_2_. The p-type metal ohmic contact was made by evaporating Pt:Ti:Pt:Au (100:400:100:1500 Å) metallization layers (bottom to top) using electron beam evaporation. The P-metal was subsequently annealed at 400°C for 1 min. We utilized a thin (10 nm) layer of AlAs that was grown on top of the bottom n-layer as an etch stop. A selective etchant was used to etch to the n-layer and was prepared by dissolving 1 *g* of citric acid monohydrate crystals in 2 ml of water. This citric acid solution (CAS) was mixed with hydrogen peroxide: 10 CAS:1 H_2_O_2_. This solution selectively etches GaAs as compared to AlGaAs layers (Kim, 1998). The chips were dipped into a solution of 1 BOE (buffered oxide etch):15 DI H_2_O for 15 s to remove the native oxide layer before etching. The AlAs layer is removed by dipping the sample into a solution of 1 BOE:15 DI H_2_O for 15 s. The n-type contact was fabricated by evaporating Ge:Au:Ge:Au:Ni:Au (60:100:100:240:100:1500 Å) metallization layers (bottom to top) and annealed at 430°C for 1 min. After contacts were made the devices were tested under a probe station to evaluate their electrical characteristics. An insulating layer of 360 nm of silicon nitride was deposited using plasma enhanced chemical vapor deposition (PECVD). Contacts were exposed by etching silicon nitride with reactive ion etching (RIE) using 50 sccm SF_6_ at 100 mTorr pressure and 150 W power. Individual devices were removed from the chips by a release procedure that consisted of creating 150 μm cuts around the devices by the DISCO automatic dicer and then backside etching with 4:1:citric acid (1 M):H_2_O_2_ (30%) at 50°C until devices separated from the chip die. **Figure 4** shows a micrograph of a fabricated shank device and the relative size of a typical FLAME device.

### OPTICAL PROBE STATION MEASUREMENTS

We analyzed the current-voltage (I–V) characteristics of the fabricated FLAME devices using standard electronic test equipment (HP 4186A semiconductor parameter analyzer) and an 856 nm laser diode under different illumination conditions. Laser light was focused onto the active area of the photodiode. BeCu coated tungsten probes (T-4-125, GCB Industries, Inc.) connected to three-axis manipulators were used to make contact with the anode and cathode of the device.

Spectral responsivity measurements were performed by focusing a tungsten white light source onto a monochromator (Acton Research Corporation) that is free-space coupled into a multi-mode fiber optic cable. The fiber-optic cable’s output was then demagnified onto the photodiode active area to measure the responsivity of the device while the monochrometer’s center wavelength was swept between 700–900 nm at 1 nm intervals. The full-width at half-maximum (FWHM) spectral resolution of the light source was 3 nm.

### LIGHT INTENSITY MEASUREMENTS IN THE RAT CORTEX AND SIMULATIONS OF WAVELENGTH SELECTIVITY

*In vivo* light intensity measurements (Abdo et al., 2013) are used here to estimate the selectivity of FLAME stimulators inside biological tissue. A brief explanation of the experimental methods is included below.

Under anesthesia, a 4 mm × 6 mm cranial opening was made rostral to the bregma on the right side of the central fissure. A 25 gage needle was slowly inserted into the brain through the base of the skull using a ventral approach, by avoiding major vessels, until it reached a few millimeters below the cortical surface. An optical fiber (diameter 100 μm, glass) was attached to a micromanipulator and inserted through the lower end of the needle until its tip was leveled with the cortical surface. A free-space laser beam (830 nm) was aimed from above to the cortex, which had a circular foot print of 0.56 mm in diameter at the dural surface. A train of optical pulses (10 ms pulse width, 25 pulses at 1 pulse per second) was applied while the light intensities were measured at depths ranging from 100 to 2500 μm from the cortical surface by the photodiode and a current amplifier connected to the other end of the optical fiber. For each depth of the fiber position, the laser beam was horizontally moved in rostro-caudal direction in steps of 100 up to 1000 μm to cover a vertical plane. Light intensities from four animals were normalized at *x* = 0 μm and *y* = 100 μm and then averaged to obtain a 2D profile of light intensities in a parasagittal plane.

Simulations of spatial and wavelength selectivity of FLAME stimulators placed in the rat cortex were executed in MATLAB. Raw light intensity data was linear interpolated. FLAME A and FLAME B devices (device areas 170 μm × 595 μm, active area 25604 μm^2^) are placed into the interpolated light intensity profile and the currents generated by the devices were calculated at each point in the *x*-*y* plane based on the measured responsivity values of the devices. The crosstalk between the devices was calculated as a function of distance between a FLAMES A and a FLAMES B device. We evaluate the effect of wavelength selectivity on the minimum implantation distance between two devices and on the maximum optical power that can be used in order to achieve selective stimulation.

## RESULTS

### ELECTRICAL CHARACTERISTICS OF FABRICATED FLAME STIMULATORS

**FIGURE 5:**
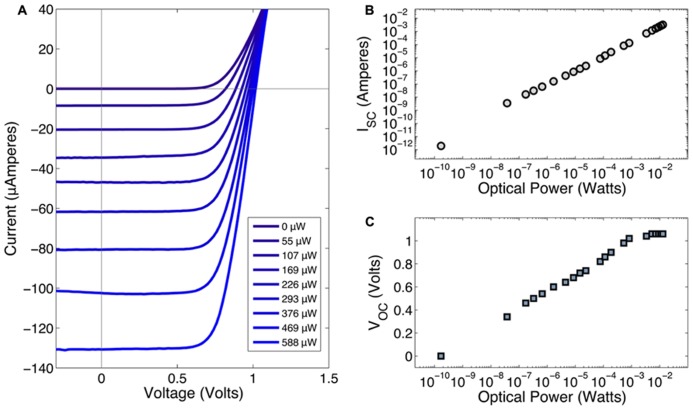
**(A)** Current-voltage characteristics of FLAME stimulators under 856 nm illumination at various optical power levels. The legend shows the optical power values in the same order as they appear in the graph. **(B)** Optical power vs. short circuit current (*I*_SC_). **(C)** Optical power vs. open-circuit voltage (*V*_OC_).

All devices showed similar I–V characteristics with an average open-circuit voltage (*V*_OC_) of 1.05 V. Data for **Figure 5** was collected from a single device using a FLAMES A device. The current-voltage (I–V) response of the device under varying optical powers (0–800 μWatts) is shown in **Figure 5A**. In **Figure 5B**, the short circuit current (*I*_SC_) is shown as a function of the optical power. These results indicate that the device has a linear response across a large range of optical powers, including those relevant for neurostimulation, i.e., 10–100 μA. **Figure 5C** shows the effect of optical power on the open-circuit voltage, *V*_OC_. As the optical power increases, *V*_OC_ also increases until it reaches saturation at 1.05 V. The conversion efficiency of the photodiode is greater than 10%. Although a properly designed photovoltaic cell should yield near-100% conversion efficiency, the presence of an optical block in our wafer structure decreases the photons received by the photodiode. Also, the lack of an anti-reflection coating further contributes to the photon loss. Dark current was 0.2 pA and the reverse bias breakdown voltage was greater than 30 V. In a previous fabrication round, we produced single GaAs/AlGaAs photodiodes that had an open-circuit voltage around 0.6 V at similar illumination levels. The FLAMES devices in this work, with two series p-i-n photodiodes, nearly doubled the open-circuit voltage as compared to the single photodiode devices providing a greater stimulation voltage at equivalent laser powers.

### SPECTRAL RESPONSIVITIES OF WAVELENGTH SELECTIVE FLAME STIMULATORS

**FIGURE 6:**
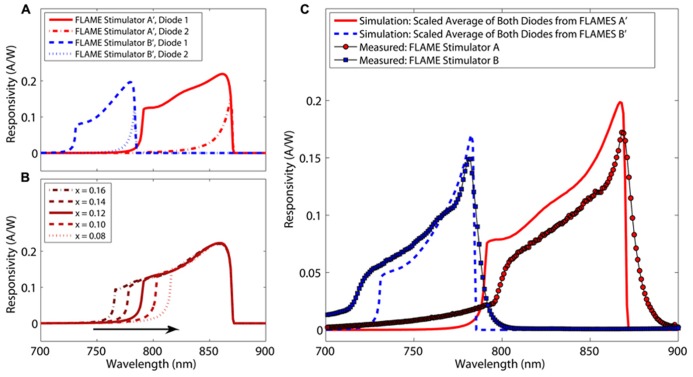
**Simulated and measured spectral responsivities of FLAME stimulators (A) Spectral responsivities of top and bottom diodes from the FLAMES A (Al_0.12_Ga_0.88_As in the optical block and GaAs in the photodiode layer) and the FLAMES B devices (Al_0.22_Ga_0.78_As in the optical block and Al_0.13_Ga_0.87_As in the photodiode layer).** **(B)** Effect of changing aluminum concentration (*x*) of optical block on the top diode of a FLAMES A device. **(C)** Measured and simulated (scaled average of bottom and top diodes) spectral responsivities shown together.

Simulated and measured spectral responsivities for FLAMES A and FLAMES B are shown in **Figure 6** (FLAMES A′ and FLAMES B′ notations are used for simulated devices). **Figure 6B** shows an example of the effect of changing aluminum concentration (*x*) of the optical block on the responsivity of the top diode in FLAMES A device. A responsivity analysis is performed for both top and bottom diodes and for both FLAME stimulators at different aluminum concentrations for the optical block and device layers. The average response from the bottom and top intrinsic layers in each diode is scaled by an additional 23%, which corresponds to the percentage of additional heavily doped regions whose current contributions are not included in the calculation by the simulation program. The best fitting data as compared to the measured responsivity curves is used as the estimated aluminum concentration of our wafers. According to these estimations, the FLAMES A device has Al_0.12_Ga_0.88_As as the optical block and GaAs as the photodiode layer, and the FLAMES B device has Al_0.22_Ga_0.78_As as the optical block and Al_0.13_Ga_0.87_As as the photodiode layer. Al_0.22_Ga_0.78_As/Al_0.13_Ga_0.87_As has a peak responsivity at 782 nm and Al_0.12_Ga_0.88_As/GaAs has a peak responsivity at 868 nm.

Scaled average responses from **Figure 6A** are plotted together with the measured spectral responsivities in **Figure 6C**. The FLAMES B device had a maximum responsivity of 0.15 A/W at 782 nm (compare to simulated device: 0.17 A/W at 782 nm) and the FLAMES A device had a maximum responsivity of 0.17 A/W at 868 nm (compare to 0.19 A/W at 866 nm). Measured responsivities are similar to the simulation responsivity values in **Figure 6C**. An 80 nm separation was achieved between the peak spectral responsivities of the two custom designs.

Although the FLAMES B device is very selective, i.e., its responsivity goes to 0.0001 A/W at 868 nm, the FLAMES A device is not as selective due to a significant responsivity (0.017 A/W) persisting at 782 nm. We will elaborate on this in the discussion section. To improve the selectivity of this device, we performed an analysis to optimize the stimulation wavelength that will be used in *in vivo* simulations in the next section. Selectivity at a given wavelength is defined as the ratio of the responsivity of the FLAMES B device to the responsivity of the FLAMES A device. In our devices, there is a tradeoff between selectivity and responsivity. A selectivity figure of merit (FOM) is used to determine the optimal wavelength (Eq. 1), where *R* is the responsivity of the device and *S* is the selectivity ratio.

(1) FOM=R*S⁢

**FIGURE 7:**
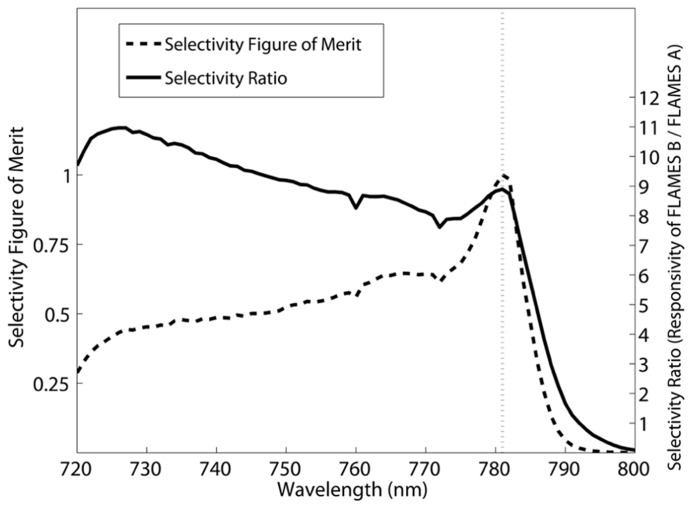
**Selection of the optimum wavelength to maximize wavelength selectivity over a range of responsivities.** Selectivity is defined to be the responsivity of the FLAMES B device divided by that of the FLAMES A device. The selectivity figure of merit (Eq. 1) and selectivity ratio are plotted. 781 nm was selected as the optimal wavelength where the selectivity figure of merit is at a maximum.

Using the FOM equation (Eq. 1), we find an optical wavelength of 781 nm to be optimal. **Figure 7** shows how selectivity changes with the illumination wavelength and the effect on the FOM.

### SPATIAL SELECTIVITY BASED ON LIGHT INTENSITY PROFILE MEASURED IN THE RAT BRAIN

**FIGURE 8:**
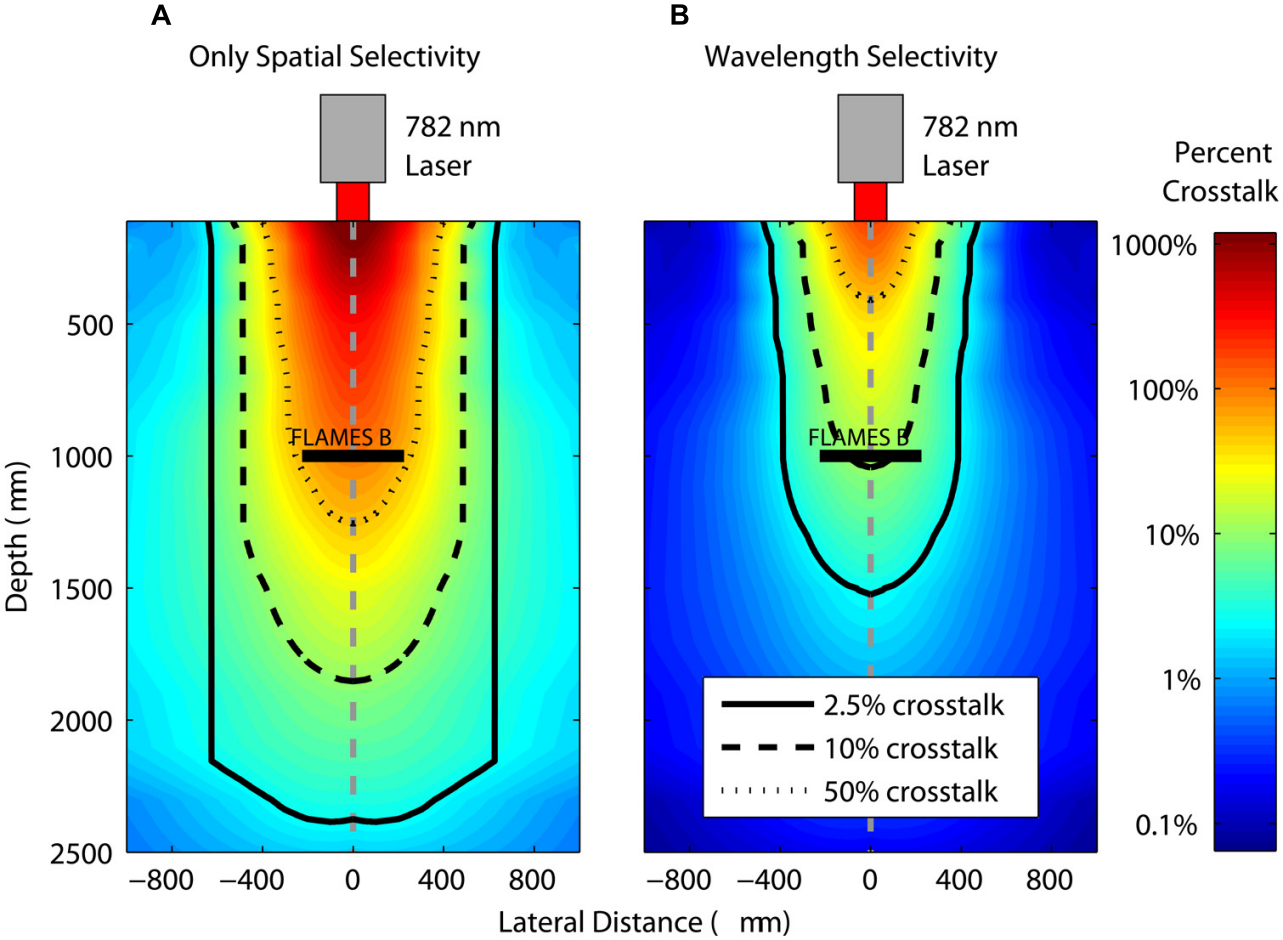
**Crosstalk percentages between FLAMES devices in the presence and absence of wavelength selectivity.** A FLAMES B device is placed in the axial center of the rat brain light intensity profile at a 1000 μm depth. In **(A)**, the profile shows the crosstalk percentages in another FLAMES B device (normalized by the current in the first FLAMES B device that is in the center). In **(B)**, the profile shows the percent crosstalk from a FLAMES A device normalized by the current of the first FLAME B device in the center. The location of pixels in the plots correspond to the center point of the second FLAMES in which the crosstalk is measured. Contour plots define the region for a certain crosstalk value between two devices: 50, 10, 2.5%.

First, we evaluate how close a second device can be placed for a given value of crosstalk between the FLAMES A and FLAMES B devices. Thus, the FLAMES B device was placed at the axial center of the light intensity profile in the rat brain at 1000 μm depth (**Figure 8**). The currents from a second device were calculated as the percentage of the current generated in the first device in the presence and absence of wavelength selectivity to quantify its contribution to spatial selectivity. Both devices were chosen to be the B type to evaluate spatial selectivity alone first (**Figure 8A**), and then the second device was made the A type to add wavelength selectivity (**Figure 8B**). Percent crosstalk is defined as the amount of current generated in the second device normalized by the current in the first FLAMES B device. **Figure 8B** shows the percent crosstalk in a FLAMES A device that is similarly moved, which demonstrates the contribution of wavelength selectivity to spatial selectivity. The effect of wavelength selectivity can be determined by comparing the contour lines corresponding to the same crosstalk percentage values (e.g., 50, 10, 2.5%) in the two plots.

**FIGURE 9:**
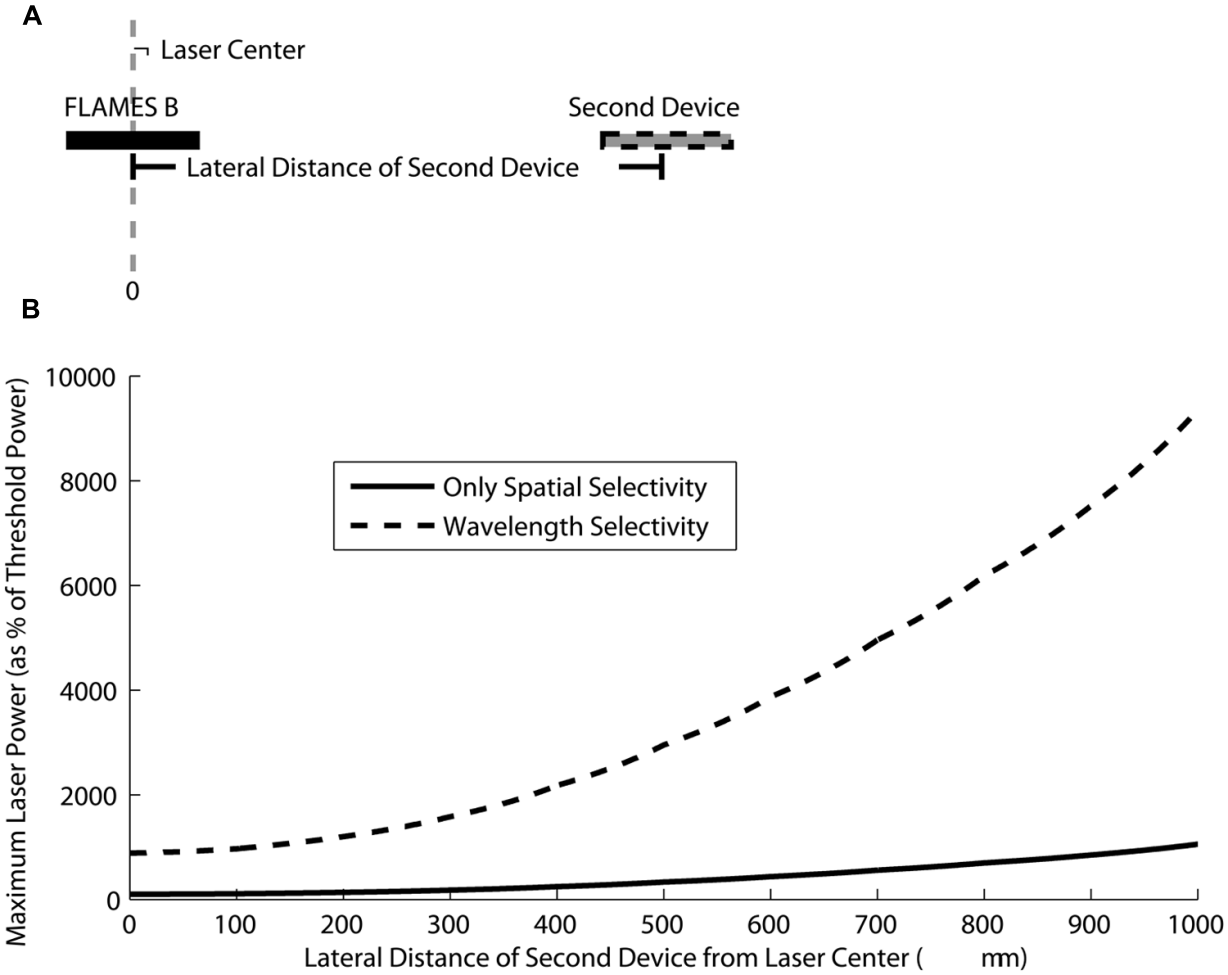
**(A)** A figure showing a FLAMES B device implanted in the optical beam center at a 1500 μm depth in the rat brain and a second device (either another FLAMES B device or a FLAMES A device) implanted at different center-to-center distances with respect to the first device. **(B)** The maximum optical power that can be tolerated before the second device is stimulated is plotted as a percentage of the threshold stimulation power (the power required to obtain a 10 μA current in the second device). When the second device is another FLAMES B device, the only source of selectivity is spatial selectivity (solid line). When the second device is a FLAMES A device, wavelength selectivity is added to spatial selectivity (dashed line). Wavelength selectivity allows the usage of much higher optical powers when compared to the case of spatial selectivity alone.

Based on the selectivity estimations in **Figure 8**, one can select a certain crosstalk value, an implantation depth, and distance between the two devices and decide on the maximum power that can be used before the second device is activated. Here, for clarity, we will present an example of selective stimulation with a FLAMES B device that is implanted at a depth of 1500 μm in the rat brain. We use 10 μA as the activation threshold, the minimum current generated in the device that will result in stimulation of the neural tissue. This activation current is on the same order of magnitude as the threshold current for stimulation of the rat spinal cord in a previous acute study with FLAME stimulators (Abdo et al., 2011). We calculate how much the optical power can be increased to generate 10 μA current in a second device (either another FLAMES B or a FLAMES A device) as the center-to-center distance between the devices increases. In other words, we calculate the maximum optical power that can be used to stimulate the FLAMES B device in the center of the optical profile and at the same time generate a current in the second device that is just below the threshold and hence not activating the surrounding neural tissue. As the distance between the devices increases, the optical power can also be increased because the current generated in the second device decreases with distance, as shown in **Figure 9**. Wavelength selectivity allows one to increase the optical power without activating the second device, thus providing better selectivity. For example, for a separation of 500 μm between the devices, the optical power can be increased by 26.5 times the threshold power without activating the second device in the presence of wavelength selectivity whereas the same value is only three times without wavelength selectivity, an improvement factor of over 8.8.

**FIGURE 10:**
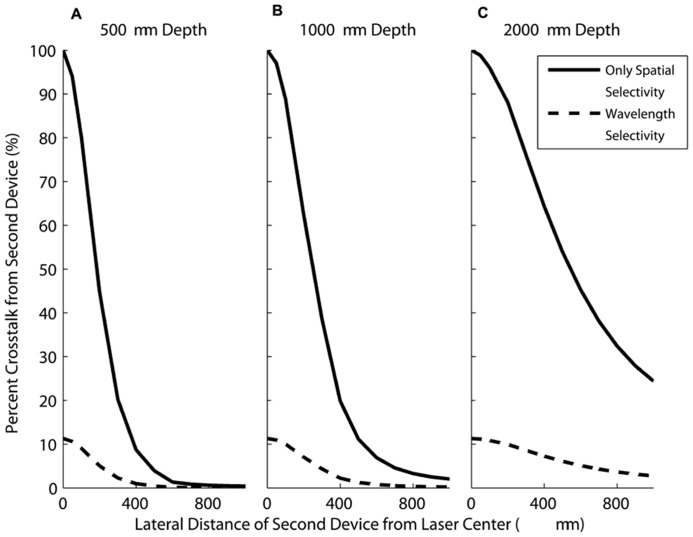
**The current generated in a second device (moving in the horizontal direction) as a percentage of the current in a FLAMES B device placed in the center when both devices are at depths of (A) 500 μm (B) 1000 μm (C) 2000 μm.** When no wavelength selectivity is assumed (solid line), the current generated in the moving device is much greater compared to devices with wavelength selectivity included (dashed line), for all depths.

**Figure 10** shows the crosstalk currents from the second device as a function of the horizontal distance from the first device at different depths. The currents generated by the second device are calculated as a percentage of the current generated by the FLAMES B device. **Figure 10** shows the percent crosstalk from the second device as a function of its distance from the first device at the depths of 500, 1000, and 2000 μm.

In each graph, both wavelength selectivity (where the second device is a FLAMES A device) and spatial selectivity (the second device is another FLAMES B device) cases are plotted. When there is only spatial selectivity, the currents generated in the second device are greater for any separation as compared to the case where there is also wavelength selectivity. For example, at a depth of 500 μm, the highest crosstalk between wavelength selective devices is 11.3%, as shown in **Figure 10A**. This occurs when the devices are placed right next to each other. As the distance between the devices increases, the crosstalk decreases. With only spatially selectivity, the crosstalk is 100% when devices are next to each other. Also, as the depth of implantation increases, the spatial selectivity decreases because the light intensity profile does not change dramatically in the horizontal direction at the deeper points. This makes wavelength selectivity more important at deeper locations. As the optical power increases, crosstalk increases linearly, therefore, one can calculate the maximum amount of power that can be used before the second device placed at a certain point is activated.

## DISCUSSION

In this study, we fabricated and evaluated wireless, wavelength selective neurostimulators based on GaAs/AlGaAs heterostructures for use in acute multisite neural implants. In many neural prosthetic applications multiple stimulation channels are required. Optical stimulation is a potential method for a selective stimulation tool through the use of different illumination wavelengths. The availability of laser diodes or LEDs at different wavelengths allows photodiodes with narrow spectral responsivities to achieve addressability.

Series diodes connected through a highly doped tunneling junction increased the open-circuit voltage to 1.05 V, providing a stimulator with usable conversion efficiencies greater than 10%. It may be argued that the device output voltage will be lower *in vivo* due to loading of neural tissue impedance around the device. Although this is true for the output voltage, the device current will not be affected by the load impedance since a photodiode acts like a current supply for device voltages that are much less than the open-circuit voltage. (The smaller the voltage, the more the device will behave like an ideal current source.) The selectivities reported here are thus independent of tissue/contact interface impedances since both device types, with and without wavelength selectivity, are assumed to have the same contact sizes and geometry. The same current injected through the same contact size produces a similar voltage field in neural tissue and hence a similar stimulus effect. A higher contact/tissue impedance, which is determined by the type of contact material used, will only increase the output voltage of the device before the contacts. The contact impedance is upper bounded by the fact that the output voltage will eventually saturate at 1.05 V and the device will begin acting like a voltage supply. Therefore, highly porous materials with low electrode/electrolyte interface impedances should be preferred for the contacts.

Light propagation through the tissue and conversion to electric current in the device is instantaneous. The stray device capacitances that result from the fabrication process have been reduced to levels that permit operational frequencies in the GHz range, suggesting sub-nanosecond delays. The contact capacitors that are in series to the path of the device current do not cause any lags in delivery of the stimulus to the tissue. Hence, speed is not a design challenge with this optical technology considering that neural stimulation takes place in tens of microseconds.

Any practical stimulus pattern and pulse waveform that is desired for neural stimulation can be implemented by modulating the intensity of the external laser source. However, the waveform of the reverse current phase, which is commonly used to discharge the contact capacitors that are charged during the forward phase, cannot be controlled. The current plan is to let the contact capacitors discharge through the leakage resistance of the device and the tissue impedance passively once the optical pulse is turned off. The RC time constant of this method was small enough to allow stimulus frequencies above 100 Hz in our *in vivo* tests (Abdo et al., 2011). The device leakage current can be increased by changing semiconductor dopings or a parallel resistor can be included in the design if shorter discharge times are needed.

We have demonstrated the concept of addressability via wavelength selectivity by fabricating two different devices, which are activated at different wavelengths: 782 and 868 nm, and simulating their response in the rat cortex. Maximum responsivities are achieved at the corresponding wavelengths for both devices. The 782 nm specific devices (FLAMES B) have a narrow spectral responsivity curve and a better fit to the simulated data than the 868 nm specific devices (FLAMES A). The latter devices have a broader spectral responsivity curve at short wavelengths and exhibit a broadening behavior that was not observed in simulations. The optical block of this device (Al_0.12_Ga_0.88_As) does not block shorter wavelengths efficiently. Addressing this deficiency is important as this response limits the wavelength selectivity of the devices. We speculate on several possibilities regarding this matter.

One explanation is the fact that on the long wavelength (low energy) side, heavily doped regions will have band narrowing and absorption will extend into the band gap. Another possibility is that the electron-hole pairs generated in the optical block at the short wavelengths are ineffectively blocked as the generated free carriers recombine, a portion of which will radiate back into the photovoltaic region. New approaches can be implemented into the wafer design to overcome this radiation. A common method to reduce the minority carrier lifetime and radiation is low temperature growth of GaAs layers (Gupta et al., 1991). By using this method, we can prevent the migration of carriers out of the optical block and into the device.

When compared to commonly used neural electrodes, the most appealing features of FLAME stimulators are their small size (<0.01 mm^3^) and wireless energy transfer method. Both of these features make FLAME stimulators a promising technology for possible use in chronic implants. Having sub-millimeter dimensions, FLAME stimulators will displace a smaller amount of tissue than the RF powered microelectrode arrays. Being optically activated, FLAME stimulators have the advantage of not having any wires, thus a reduction in chronic tissue response and failures associated with wire breakage that are expected. Wavelength selectivity of FLAME stimulators should allow implantation of multiple devices at a higher spatial density by decreasing the crosstalk between the devices. The maximum optical power calculated in simulations is many times larger than the threshold power for activation of the primary device. Such a large margin of tolerance between the primary and secondary device may seem to be unnecessary because in most neural stimulation applications the recruitment curve vs. current amplitude is very steep and therefore the supra maximal activation is achieved quickly above the threshold current. However, activation of the primary device at power levels several times higher than the supra maximal level may be needed due to the fact that the implanted stimulators will move along with the neural tissue with respect to the optical fiber delivering the light power. We envision attaching the fiber optic to some bony structure outside the CNS for chronic applications (Sahin and Pikov, 2011). Thus the devices may move in and out of focus when the spinal cord or the brain moves with respect to the vertebrae or the skull, respectively. The wavelength selectivity will be of particular importance when the translation of the tissue will be in the same order as the distance between the devices. In this case, the spatial selectivity will be reduced to wavelength selectivity. According to **Figure 9**, the wavelength selectivity alone will provide about eight times of threshold difference (for zero distance) between the targeted and the nearby FLAME stimulator.

## Conflict of Interest Statement

The authors declare that the research was conducted in the absence of any commercial or financial relationships that could be construed as a potential conflict of interest.
